# Supplementary material for: Occurrence of Plasmid-Mediated Quinolone Resistance and Carbapenemase-Encoding Genes in Pseudomonas aeruginosa Isolates from Nosocomial Patients in Aguascalientes, Mexico
Source: Pathogens. 2024 Nov 13;13(11):992. doi: 10.3390/pathogens13110992 (PMC11597332; doi:10.3390/pathogens13110992)
Supplement: Supplementary file 1 [file pathogens-13-00992-s001.zip › pathogens-3281398-supplementary.pdf]

Supplementary Table S1. Oligonucleotides used in this study.

Oligonucleotide name	Primer sequence 5′→3	Amplicon size (bp)	Anneling temperature (°C)	Reference
***Pseudomonas* sp., detection**				
PA-GS	GACGGGTGAGTAATGCCTA<br>CACTGGTGTTCCTTCCTATA	618	51	[33]
**Carbapenemase-encoding genes**				
bla_{OXA-48}	ATTATCGGAATGCCTGCGGT<br>CGTCGAGCCAGAAACTGTCT	427	53	This study
bla_{VIM}	GATGGTGTTTGGTCGCATA<br>CGAATGCGCAGCACCAG	389	53	This study
bla_{OXA-51}	TTTTGGCTGGTGGGTCCTTT<br>GAGGCTGAACAACCCATCCA	223	53	This study
bla_{NDM}	ATATTATGCACCCGGTCGCG<br>TCATCACGATCATGCTGGCC	720	53	This study
bla_{KPC}	GCCGTCTAGTTCTGCTGTCT<br>CGCTGTGCTTGTCATCCTTG	800	55	This study
bla_{OXA-1}	GCAGCGCCAGTGCATCAAC<br>CCGCATCAAATGCCATAAGTG	420	51	[37]
bla_{OXA-23}	TTCTGGTTGTACGGTTCAGCA<br>TGCCCAACCAGTCTTTCCAA	600	53	This study
bla_{IMP}	TCACTTGGTTTGTGGAGCGT<br>TTTGCGTCACCCAAATTGCC	336	54	This study
bla_{GES}	CTCAGATCGGTGTTGCGATC<br>TGTATCTCTGAGGTCGCCAG	416	53	[38]
**Plasmid-mediated quinolone resistance genes**				
qnrA	TCAGCAAGAGGATTTCTCA<br>GGCAGCACTATTACTCCCA	605	46	[41]
qnrB	GATCGTGAAAGCCAGAAAGG<br>ACGATGCCTGGTAGTTGTCC	469	51	[40]
qnrC	GGGTTGTACATTTATTGAATC<br>TCCACTTTACGAGGTTCT	447	46	[41]
qnrS	ACGACATTCGTCAACTGCAA<br>TAAATTGGCACCCTGTAGGC	417	51	[40]
qnrD	CGAGATCAATTTACGGGGAATA<br>AACAAGCTGAAGCGCCTG	582	51	[42]
aac-(6´)-lb	TTGCGATGCTCTATGAGTGGCTA<br>CTCGAATGCCTGGCGTGTTT	482	55	[43]
oqxA	GACAGCGTCGCACAGAATG<br>GGAGACGAGGTTGGTATGGA	339	55	[44]
**Plasmid-mediated colistin resistance**				
mcr-1	GCAACCAAGCCTGATATGCG<br>GCTTAAAATACGCAGGCCCG	519	55	This study

Supplementary Table S2. Breakpoint values of the antimicrobial agents used in this study.

Antimicrobial group	Antimicrobial agent	*MIC breakpoints (µg/mL)		
		**S**	**I**	**R**
Beta-lactam combination agents	Piperacillin-tazobactam	≤ 16/4	32/64-4	≥ 128/4
Cephalosporins	Ceftazidime	≤ 8	16	≥ 32
	Cefepime	≤ 8	16	≥ 32
	Ceftriaxone	≤ 8	16	≥ 32
Carbapenems	Doripenem	≤ 2	4	≥ 8
	Imipenem	≤ 2	4	≥ 8
	Meropenem	≤ 2	4	≥ 8
Lipopeptides	Colistin	≤ 0.5	≤ 2	≥ 4
Aminoglycosides	Gentamicin	≤ 4	8	≥ 16
	Amikacin	≤ 16	32	≥ 64
Fluoroquinolones	Ciprofloxacin	≤ 0.5	1	≥ 2
Glycylcycline	Tigecycline	≤ 2	4	≥ 8

*MIC values are based on the CLSI document M100-ED30 [34].

Supplementary Table S3. Antimicrobial susceptibility of carbapenem-resistant *P. aeruginosa* isolates.

Strain ID	CIP	IMI	MEM	DOR	COL	TG	TZP	CEF	CAZ	CRO	AMK	GEN
PHH1	≥ 4	≥ 16	≥ 16	≥ 8	2	≥ 8	16	4	4	≥ 64	≤ 2	2
PHH2	2	≥ 16	≥ 16	≥ 8	Nd	≥ 8	16	≥ 64	≥ 64	≥ 64	≥ 64	≥ 16
PHH3	≥ 4	Nd	≥ 16	Nd	2	Nd	Nd	32	≥ 64	≥ 64	≥ 64	≥ 16
PHH4	≥ 4	≥ 16	≥ 16	≥ 8	≤ 0.5	≥ 8	≥ 128	32	≥ 64	≥ 64	4	≥ 16
PHH5	≥ 4	Nd	≥ 16	≥ 8	≤ 0.5	≥ 8	Nd	32	≥ 64	≥ 64	≥ 64	≥ 16
PHH6	≤ 0.25	8	Nd	≥ 8	2	≥ 8	≥ 128	8	32	≥ 64	4	≤ 1
PHH7	≥ 4	≥ 16	≥ 16	≥ 8	2	≥ 8	64	≥ 64	≥ 64	≥ 64	≥ 64	≥ 16
PHH8	≥ 4	≥ 16	≥ 16	≥ 8	≤ 0.5	≥ 8	≥ 128	≥ 64	≥ 64	≥ 64	≥ 64	≥ 16
PHH9	≥ 4	≥ 16	8	≥ 8	2	≥ 8	≥ 128	4	4	≥ 64	≥ 64	8
PHH10	0.5	≥ 16	≥ 16	≥ 8	1	≥ 8	32	8	8	≥ 64	≤ 2	≤ 1
PHH11	≥ 4	≥ 16	≥ 16	≥ 8	≤ 0.5	≥ 8	64	16	16	≥ 64	8	8
PHH12	≥ 4	≥ 16	≥ 16	≥ 8	≤ 0.5	≥ 8	32	16	16	≥ 64	8	8
PHH13	0.5	≥ 16	≥ 16	≥ 8	≥ 16	≥ 8	≥ 128	8	32	≥ 64	≤ 2	≤ 1
PHH14	≥ 4	Nd	≥ 16	Nd	Nd	Nd	Nd	≥ 64	≥ 64	≥ 64	≥ 64	≥ 16
PHH15	≥ 4	≥ 16	≥ 16	≥ 8	2	≥ 8	≥ 128	≥ 64	≥ 64	≥ 64	≥ 64	≥ 16
PHH16	≥ 4	≥ 16	≥ 16	≥ 8	≤ 0.5	≥ 8	≥ 128	≥ 64	≥ 64	≥ 64	≥ 64	≥ 16
PHH17	≥ 4	≥ 16	≥ 16	≥ 8	≤ 0.5	≥ 8	≥ 128	≥ 64	≥ 64	≥ 64	≥ 64	≥ 16
PHH18	≥ 4	≥ 16	≥ 16	≥ 8	≤ 0.5	≥ 8	16	8	32	≥ 64	≥ 64	≥ 16
PHH19	≥ 4	≥ 16	≥ 16	≥ 8	≤ 0.5	≥ 8	≥ 128	≥ 64	≥ 64	≥ 64	≥ 64	≥ 16
PHH20	≥ 4	≥ 16	≥ 16	≥ 8	Nd	≥ 8	≥ 128	≥ 64	≥ 64	≥ 64	≥ 64	≥ 16
PHH21	≥ 4	≥ 16	≥ 16	≥ 8	≤ 0.5	≥ 8	≥ 128	≥ 64	≥ 64	≥ 64	≥ 64	≥ 16
PHH22	> 2	> 4	> 8	Nd	Nd	Nd	> 64	>16	> 16	> 32	≥ 32	> 8
PHH23	0.5	≥ 16	≥ 16	≥ 8	≤ 0.5	≥ 8	32	8	16	≥ 64	4	≤ 1
PHH24	≥ 4	≥ 16	≥ 16	≥ 8	≤ 0.5	Nd	≥ 128	≥ 64	≥ 64	Nd	≥ 64	≥ 16
PHH25	≥ 4	Nd	≥ 16	Nd	Nd	Nd	Nd	≥ 64	≥ 64	Nd	≥ 64	≥ 16
PHH26	0.5	≥ 16	≥ 16	≥ 8	≤ 0.5	≥ 8	32	8	16	Nd	≤ 2	≤ 1
PHH27	≥ 4	≥ 16	≥ 16	≥ 8	≤ 0.5	≥ 8	32	≥ 64	≥ 64	≥ 64	≥ 64	4
PHH28	≥ 4	≥ 16	≥ 16	≥ 8	≤ 0.5	≥ 8	≥ 128	≥ 64	≥ 64	≥ 64	≥ 64	≥ 16
PHH29	≥ 4	≥ 16	≥ 16	≥ 8	≤ 0.5	≥ 8	≥ 128	≥ 64	≥ 64	≥ 64	≥ 64	≥ 16
PHH30	≥ 4	≥ 16	≥ 16	≥ 8	≤ 0.5	≥ 8	≥ 128	≥ 64	Nd	≥ 64	≥ 64	≥ 16
PHH31	≥ 4	Nd	≥ 16	Nd	Nd	Nd	Nd	≥ 64	Nd	≥ 64	≥ 64	≥ 16
PHH32	2	Nd	≥ 16	Nd	Nd	Nd	Nd	8	Nd	≥ 64	≤ 2	≤ 1
PHH33	≥ 4	≥ 16	≥ 16	≥ 8	≤ 0.5	≥ 8	Nd	≥ 64	≥ 64	≥ 64	≥ 64	≥ 16
PHH34	≥ 4	≥ 16	≥ 16	≥ 8	≤ 1	≥ 8	≥ 128	≥ 64	≥ 64	≥ 64	≥ 64	≥ 16
PHH35	≥ 4	Nd	≥ 16	Nd	2	Nd	Nd	32	≥ 64	≥ 64	≥ 64	≥ 16
PHH36	≥ 4	≥ 16	≥ 16	≥ 8	2	≥ 8	≥ 128	32	≥ 64	≥ 64	≥ 64	≥ 16
PHH37	≥ 4	≥ 16	≥ 16	≥ 8	≤ 0.5	≥ 8	≥ 128	32	≥ 64	≥ 64	≥ 64	≥ 16
PHH38	0.5	≥ 16	≥ 16	≥ 8	≤ 0.5	≥ 8	Nd	8	4	≥ 64	≤ 2	3
PHH39	≥ 4	≥ 16	≥ 16	≥ 8	≤ 0.5	≥ 8	64	≥ 64	≥ 64	≥ 64	≥ 64	≥ 16
PHH40	≥ 4	≥ 16	≥ 16	≥ 8	≤ 0.5	≥ 8	Nd	≥ 64	≥ 64	≥ 64	≥ 64	≥ 16

CIP: ciprofloxacin, IMI: imipenem, MEM: meropenem, DOR: doripenem, COL: colistin, TG: tigecycline, TZP: peracillin/tazobactam, CEF: cefepime, CAZ: ceftazidime, CRO: ceftriaxone, AMK: amikacin, GEN: gentamicin; Nd: non-determined.

Supplementary Table S3. Antimicrobial susceptibility of carbapenem-resistant *P. aeruginosa* strains (continuation).

Strain ID	CIP	IMI	MEM	DOR	COL	TG	TZP	CEF	CAZ	CRO	AMK	GEN
PHH41	≥ 4	≥ 16	≥ 16	≥ 8	≤ 0.5	≥ 8	Nd	≥ 64	≥ 64	≥ 64	≥ 64	≥ 16
PHH42	≥ 4	≥ 16	≥ 16	≥ 8	≤ 0.5	≥ 8	≥ 128	≥ 64	≥ 64	≥ 64	≥ 64	≥ 16
PHH43	≥ 4	≥ 16	≥ 16	≥ 8	≤ 0.5	≥ 8	≥ 128	≥ 64	≥ 64	≥ 64	≥ 64	≥ 16
PHH44	≥ 4	≥ 16	≥ 16	≥ 8	≤ 0.5	Nd	≥ 128	32	Nd	≥ 64	≥ 64	≥ 16
PHH45	≥ 4	Nd	≥ 16	Nd	Nd	Nd	Nd	16	32	≥ 64	≥ 64	≥ 16
PHH48	≥ 4	≥ 16	≥ 16	≥ 8	≤ 0.5	≥ 8	≥ 128	≥ 64	≥ 64	≥ 64	≥ 64	≥ 16
PHH49	≥ 4	≥ 16	≥ 16	≥ 8	≤ 0.5	≥ 8	Nd	≥ 64	≥ 64	≥ 64	≥ 64	≥ 16
PHH50	≥ 4	≥ 16	≥ 16	≥ 8	≤ 0.5	≥ 8	≥ 128	≥ 64	≥ 64	≥ 64	≥ 64	≥ 16
PHH51	≥ 4	≥ 16	≥ 16	≥ 8	1	≥ 8	≥ 128	≥ 64	≥ 64	≥ 64	≥ 64	≥ 16
PHH52	≥ 4	≥ 16	≥ 16	≥ 8	4	≥ 8	≥ 128	≥ 64	≥ 64	≥ 64	≥ 64	≥ 16
PHH53	≥ 4	≥ 16	≥ 16	≥ 8	≤ 0.5	≥ 8	≥ 128	≥ 64	≥ 64	≥ 64	4	8
PHH54	≥ 4	≥ 16	≥ 16	≥ 8	≤ 0.5	≥ 8	≥ 128	≥ 64	≥ 64	≥ 64	≥ 64	≥ 16

CIP: ciprofloxacin, IMI: imipenem, MEM: meropenem, DOR: doripenem, COL: colistin, TG: tigecycline, TZP: peracillin/tazobactam, CEF: cefepime, CAZ: ceftazidime, CRO: ceftriaxone, AMK: amikacin, GEN: gentamicin; Nd: non-determined.

Supplementary Table S4. Carbapenemase-encoding and Plasmid-mediated quinolone resistant genes in *P. aeruginosa*.

ID	CIP-MIC values (µg/mL)	CIP-susceptibility interpretation	Carbapenemase-encoding genes			PMQR genes			PMCR
			MBL	OXA	SBL	Qnr variants	Efflux pumps	Aminoglycoside variant	*mcr-1*
PHH1	≥ 4	R	bla_{IMP}	bla_{OXA-51}		*qnrC*	*oqxA*	*aac-(6´)-lb*	
PHH2	2	R		bla_{OXA-51}				*aac-(6´)-lb*	
PHH3	≥ 4	R		bla_{OXA-51}			*oqxA*		
PHH4	≥ 4	R		bla_{OXA-51}				*aac-(6´)-lb*	
PHH5	≥ 4	R	bla_{VIM}	bla_{OXA-51}				*aac-(6´)-lb*	
PHH6	≤ 0.25	S	bla_{VIM}	bla_{OXA-51}			*oqxA*		
PHH7	≥ 4	R	bla_{VIM}		bla_{KPC}	*qnrB, qnrC, qnrS*	*oqxA*		
PHH8	≥ 4	R		bla_{OXA-51}					
PHH9	≥ 4	R				*qnrC, qnrS*	*oqxA*		
PHH10	0.5	S				*qnrC, qnrS*	*oqxA*		
PHH11	≥ 4	R				*qnrC, qnrS*	*oqxA*		
PHH12	≥ 4	R	bla_{VIM}	bla_{OXA-48}		*qnrS*			*mcr-1*
PHH13	0.5	S		bla_{OXA-48}	bla_{KPC}	*qnrS*	*oqxA*		
PHH14	≥ 4	R				*qnrC*			
PHH15	≥ 4	R						*aac-(6´)-lb*	
PHH16	≥ 4	R		bla_{OXA-51}			*oqxA*		
PHH17	≥ 4	R							
PHH18	≥ 4	R		bla_{OXA-51}, bla_{OXA-1}			*oqxA*		
PHH19	≥ 4	R		bla_{OXA-51}, bla_{OXA-1}			*oqxA*	*aac-(6´)-lb*	

CIP: ciprofloxacin. MIC values ≥ 4 represent resistant to ciprofloxacin, MIC values ≤ 0.5 represent susceptibility to ciprofloxacin.

Supplementary Table S4. Carbapenemase-encoding and Plasmid-mediated quinolone resistant genes in *P. aeruginosa*. (continuation).

ID	CIP-MIC values (µg/mL)	CIP-susceptibility interpretation	Carbapenemase-encoding genes			PMQR genes			PMCR
			MBL	OXA	SBL	Qnr variants	Efflux pumps	Aminoglycoside variant	*mcr-1*
PHH20	≥ 4	R	bla_{VIM}	bla_{OXA-51}, bla_{OXA-1}					
PHH21	≥ 4	R	bla_{VIM}	bla_{OXA-51}					
PHH22	> 2	R		bla_{OXA-51}			*oqxA*		
PHH23	0.5	S					*oqxA*		
PHH24	≥ 4	R		bla_{OXA-1}					
PHH25	≥ 4	R		bla_{OXA-51}					
PHH26	0.5	S	bla_{VIM}						
PHH27	≥ 4	R		bla_{OXA-51}		*qnrC*	*oqxA*		
PHH28	≥ 4	R							
PHH29	≥ 4	R		bla_{OXA-48}		*qnrS*	*oqxA*		
PHH30	≥ 4	R					*oqxA*		
PHH31	≥ 4	R	bla_{IMP}	bla_{OXA-51}					
PHH32	2	R		bla_{OXA-51}					
PHH33	≥ 4	R		bla_{OXA-51}	bla_{KPC}		*oqxA*		
PHH34	≥ 4	R		bla_{OXA-51}, bla_{OXA-48}					
PHH35	≥ 4	R		bla_{OXA-51}					
PHH36	≥ 4	R					*oqxA*		
PHH37	≥ 4	R					*oqxA*		
PHH38	0.5	S			bla_{GES}	*qnrB*, *qnrS*	*oqxA*		
PHH39	≥ 4	R					*oqxA*	*aac-(6´)-lb*	
PHH40	≥ 4	R	bla_{NDM}				*oqxA*		

CIP: ciprofloxacin. MIC values ≥ 4 represent resistant to ciprofloxacin, MIC values ≤ 0.5 represent susceptibility to ciprofloxacin.

Supplementary Table S4. Carbapenemase-encoding and Plasmid-mediated quinolone resistant genes in *P. aeruginosa* (continuation).

ID	CIP-MIC values (µg/mL)	CIP-susceptibility interpretation	Carbapenemase-encoding genes			PMQR genes			PMCR
			MBL	OXA	SBL	Qnr variants	Efflux pumps	Aminoglycoside variant	*mcr-1*
PHH41	≥ 4	R							
PHH42	≥ 4	R		bla_{OXA-1}					
PHH43	≥ 4	R		bla_{OXA-1}		*qnrS*		*aac-(6´)-lb*	
PHH44	≥ 4	R	bla_{NDM}	bla_{OXA-1}				*aac-(6´)-lb*	
PHH45	≥ 4	R	bla_{NDM}	bla_{OXA-1}					
PHH48	≥ 4	R		bla_{OXA-51}			*oqxA*		
PHH49	≥ 4	R		bla_{OXA-51}			*oqxA*	*aac-(6´)-lb*	
PHH50	≥ 4	R						*aac-(6´)-lb*	
PHH51	≥ 4	R	bla_{VIM}					*aac-(6´)-lb*	
PHH52	≥ 4	R							
PHH53	≥ 4	R						*aac-(6´)-lb*	
PHH54	≥ 4	R							

CIP: ciprofloxacin. MIC values ≥ 4 represent resistant to ciprofloxacin, MIC values ≤ 0.5 represent susceptibility to ciprofloxacin.
